# The complete chloroplast genome sequence of the *Taraxacum officinale* F.H.Wigg (Asteraceae)

**DOI:** 10.1080/23802359.2016.1155425

**Published:** 2016-03-28

**Authors:** Jin-Kyung Kim, Jee Young Park, Yun Sun Lee, Hyun Oh Lee, Hyun-Seung Park, Sang-Choon Lee, Jung Hwa Kang, Taek Joo Lee, Sang Hyun Sung, Tae-Jin Yang

**Affiliations:** a Department of Plant Science, Plant Genomics and Breeding Institute, and Research Institute of Agriculture and Life Sciences, College of Agriculture and Life Sciences, Seoul National University, Seoul, Republic of Korea;; b Phyzen Genomics Institute, Gwanak Century Tower, Gwanak-gu, Seoul, Republic of Korea;; c Hantaek Botanical Garden, Yongin, Gyeonggi-Do, Republic of Korea;; d College of Pharmacy and Research Institute of Pharmaceutical Science, Seoul National University, Seoul, Republic of Korea;; e Crop Biotechnology Institute/GreenBio Science and Technology, Seoul National University, Pyeongchang, Republic of Korea

**Keywords:** Chloroplast, genome sequence, medicinal plant, *Taraxacum officinale*

## Abstract

*Taraxacum officinale* is a distributed weedy plant used as a traditional medicinal herb belonging to the family Asteraceae. The complete chloroplast genome of *T. officinale* was generated by *de novo* assembly with whole genome sequence data. The chloroplast genome was 151 324 bp in length, which consisted of a large single copy region of 83 895 bp and a short single copy region of 18 549 bp separated by a pair of inverted repeat regions of 24 440 bp. The chloroplast genome contained 79 protein-coding genes, 29 tRNA genes and four rRNA genes. Phylogenetic analysis revealed that *T. officinale* was closely related to *Lactuca sativa*.

The genus *Taraxacum*, known as dandelion, belongs to the family Asteraceae and widely distributed in the temperate areas of the Northern Hemisphere. More than 2500 species are known in the genus *Taraxacum* (Richards 1973; Ge et al. 2007). *Taraxacum* species have long been utilized for medicinal purposes due to their various pharmacological activities including diuretic and anti-inflammatory effects. *Taraxacum* roots include various sesquiterpens, triterpenes, phytosterols, flavonoids and phenolic compounds (Schütz et al. 2006). The genus *Taraxacum* shows taxonomic complexity due to the presence of agamospermy, complex hybrids and polyploidy, which makes taxonomic studies of this genus difficult (Ge et al. 2007). So far, complete chloroplast genome sequences of many species in the family Asteraceae have been reported including *Helianthus annuus* and *Artemisia frigida* (Timme et al. 2007; Liu et al. 2013), whereas none of *Taraxacum* species was subject to study on complete chloroplast genome sequence. On this account, we generated the complete chloroplast genome sequence of *T. officinale* for the first time in the genus *Taraxacum*. The sequence will provide the information for phylogenetic analysis and molecular study of this species as well as others in the family Asteraceae.

Total genomic DNA was extracted from leaves of *T. officinale* collected from Hantaek Botanical Garden (http://www.hantaek.co.kr), Yongin, Korea, using a modified cetyltrimethylammonium bromide protocol (Allen et al. 2006). A paired-end (PE) library with 300-bp insert size was constructed and sequenced using an Illumina MiSeq instrument (Illumina, San Diego, CA) by Lab Genomics Co. (http://www.labgenomics.co.kr), Seoul, Korea. Whole genome sequence data of 1.3 Gb were generated, trimmed, and assembled by a CLC genome assembler (v. beta 4.6, CLC Inc., Aarhus, Denmark), as described in Kim et al. (2015a,b). The representative chloroplast contigs were retrieved, ordered and combined into a single draft sequence, through comparison with the chloroplast sequence of *Panax ginseng* (GenBank accession no. NC_006290) as a guidance. The draft sequence was validated and manually corrected by PE read mapping. The protein-coding, tRNA and rRNA genes in the chloroplast genome were annotated using the DOGMA program (Wyman et al. 2004) and manual curation based on BLAST searches.

The complete chloroplast genome of *T. officinale* (GenBank accession no. KU361241) was 151 324 bp in length and showed a typical quadripartite chloroplast genome structure consisting of a large single copy region of 83 895 bp, a short single copy region of 18 549 bp, and a pair of inverted repeats regions of 24 440 bp. The chloroplast genome contained a total of 112 genes including 79 protein-coding genes, 29 tRNA genes and 4 rRNA genes. The GC content of the whole chloroplast genome was 37.7%.

Phylogenetic analysis was conducted with common 68 chloroplast protein coding sequences of *T. officinale* and other 14 species belonging to the family Asteraceae, using a maximum likelihood analysis of MEGA 6.0 (Tamura et al. 2013) with 1000 bootstrap replicates. The phylogenetic tree indicated that *T. officinale* was most closely related to *Lactuca sativa* belonging to the subfamily Cichorioideae, as expected (Figure 1).

**Figure 1.**
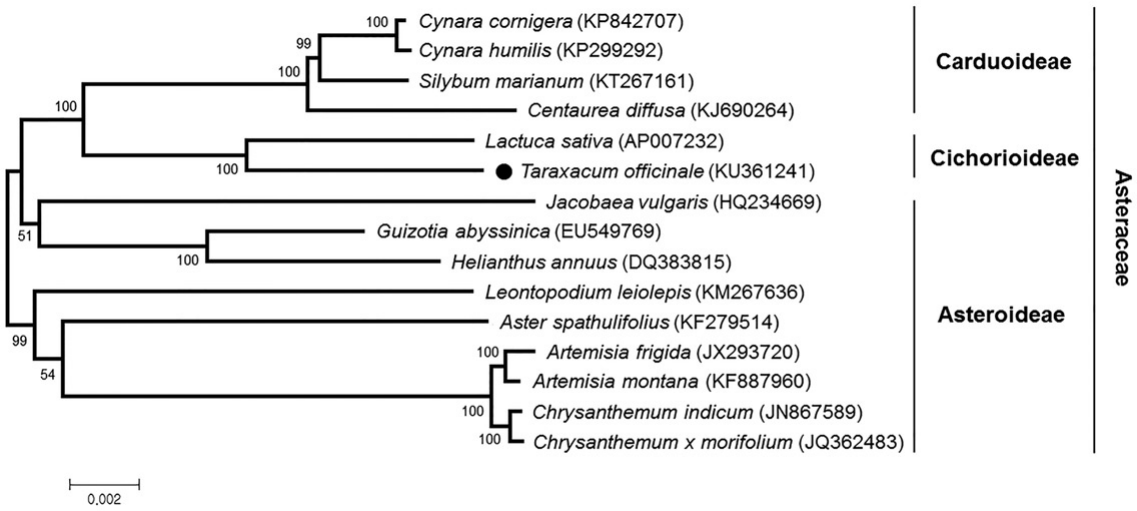
Maximum likelihood phylogenetic tree of *T. officinale* with related 14 species of the family Asteraceae based on common 68 chloroplast protein-coding genes. Numbers in the nodes are the bootstrap values from 1000 replicates.
